# Impact of body mass index at diagnosis on outcomes of pediatric acute leukemia: A systematic review and meta-analysis

**DOI:** 10.1371/journal.pone.0302879

**Published:** 2024-05-06

**Authors:** Ling Dong, Guixing Dai, Jing Zhao

**Affiliations:** Department of Pediatrics, Changxing People’s Hospital, Huzhou City, Zhejiang Province, China; Johns Hopkins University Bloomberg School of Public Health, UNITED STATES

## Abstract

**Background:**

The incidence of childhood malnutrition i.e., both obesity and undernutrition, is on a rise. While there is extensive evidence of the influence of body mass index (BMI) on the survival and other important outcomes of adult cancers, the impact of childhood BMI on one of the common pediatric cancers i.e., leukemia is not well studied.

**Methods:**

Systematic search of PubMed, Scopus, and Google Scholar databases was done to identify studies that were conducted among pediatric patients with leukemia and had examined outcomes of interest based on BMI at the time of diagnosis.

**Results:**

Effect sizes were reported as pooled hazards ratio (HR) along with 95% confidence intervals (CI). A total of 17 studies were included. Compared to pediatric leukemia patients with normal BMI, underweight (HR 1.07, 95% CI: 1.04, 1.11) and obese (HR 1.42, 95% CI: 1.18, 1.71) children with leukemia had higher risks of overall mortality. Underweight (HR 1.10, 95% CI: 1.02, 1.19) and obese (HR 1.34, 95% CI: 1.15, 1.55) pediatric leukemia patients had a tendency to lower event-free survival compared to children with normal BMI. The risk of relapse was not significant for underweight, overweight, and obese children.

**Conclusions:**

Both underweight and obese status at the time of diagnosis were associated with poor survival outcomes in pediatric patients with leukemia.

## Introduction

The incidence of childhood obesity is on the rise in the developed countries [1]. Concurrently, in many of the countries, there is a dual burden of childhood undernutrition as well as obesity [2–4]. According to a recent global estimate, around 40 million children under five years of age are either overweight or obese [5]. On the other hand, around 45–50 million children, particularly from low- and middle-income countries suffer from wasting due to severe undernutrition [6, 7].

Leukemia is one of the most prevalent childhood cancers, and one of the leading causes of cancer-related deaths among children [8–10]. Acute lymphoblastic leukaemia (ALL) accounts for around three-fourth of all pediatric leukemia cases and is currently associated with significantly improved prognosis (5-year survival of around 80–85%) [11, 12].

Malnutrition i.e., both under and over-nutrition, has been shown to influence the outcomes in adult cancers. Obesity, for instance, has been shown to influence the survival and relapse of some adult cancers [13–15]. A credible body of evidence suggests a link between cancer prognosis and inflammation due to obesity. Adiposity that results from obesity creates a pro-inflammatory environment that could possibly contribute to adverse prognostic outcomes [16]. Obesity is associated with an increased circulating levels of inflammatory cytokines such as interleukin-6 (IL-6) and tumour necrosis factor- alpha (TNF- α) that may trigger an increase in the number of cells that have tumour-forming potential [16, 17]. On the other hand, studies demonstrate that undernutrition is associated with increased tumour aggressiveness, thereby, increasing the risk of recurrence and metastasis [17, 18]. Also, some evidence from certain adult cancers suggests that undernutrition may be associated with sub-optimal response to chemotherapy and associated side effects [19, 20].

While there is evidence of the impact of body mass index (BMI) on the outcomes of certain cancers such as bladder cancer, colon cancer, nasopharyngeal cancer etc, the impact of childhood BMI on leukemia is unclear. Two prior systematic reviews on this issue have been published [21, 22]. One review by Amankwah et al. pooled 11 studies and documented that pediatric patients with high BMI were at increased risk of mortality [21]. Similar review by Orgel et al was published in the year 2016 and demonstrated that high BMI correlated with increased risk of poor event free survival (EFS) and overall survival in children with both ALL and acute myelogenous leukemia (AML) [22]. However, both these reviews were conducted nearly half a decade ago and with the new studies on this issue being published, there is a need to update the evidence.

This study aims to assess the impact of malnutrition status on the outcomes of pediatric patients with leukemia.

## Materials and methods

### Search strategy, selection criteria and relevant methods

The PRISMA guidelines were followed for conducting this meta-analysis [23]. The study was registered at PROSPERO, with the number: CRD42024497221. A systematic search was conducted using structured strategy (S1 Table) across PubMed, Scopus and Google Scholar databases. This search aimed to identify English-language studies published until 15^th^ December 2023 that examined the association between body mass index (BMI) at the time of diagnosis with overall survival, event free survival (EFS), and relapse rate in pediatric patients with leukemia.

After executing the search strategy, and deduplication, two study authors independently reviewed titles and abstracts to identify potentially relevant studies. Full texts of the studies were then read to finally select the studies for the meta-analysis. If any disagreements arose, the two authors of the study reached a shared consensus through discussion.

Eligible studies should have examined pediatric patients with confirmed leukemia diagnosis and assessed relevant outcomes based on BMI at the time of diagnosis. Standardized BMI cut-offs for pediatric populations are not universally agreed upon, and variations exist in defining categories such as underweight, normal weight, overweight, and obesity. We made a decision to combine studies with different BMI cut-offs to maximize the available data, enhance statistical power, and acknowledge the variability in BMI categorization methods across the literature. We aimed to include observational studies (either with cohort, cross-sectional or case-control design). All included studies should have reported data of the control group of pediatric leukemia patients with normal BMI.

Case reports or reviews were excluded. Also, studies that did not compare outcomes of interest based on BMI at the time of diagnosis or the outcomes reported were not relevant for this meta-analysis were excluded.

A pilot-tested data extraction sheet was employed for the extraction process. Two study authors independently gathered required data from the included studies. All disagreements were resolved by consensus. The assessment of the included studies was conducted using the Newcastle-Ottawa Quality Assessment Scale for non-randomised studies [24].

### Statistical analysis

All the analyses were done using STATA version 16.0. The effect sizes were reported as pooled hazards ratio (HR) along with 95% confidence intervals (CI). Pooled effect sizes were presented for underweight, overweight, and obese categories, compared to normal BMI at the time of diagnosis. A subgroup analysis was conducted based on diagnosis of acute lymphoblastic leukemia (ALL) or acute myeloid leukemia (AML). We used I^2^ to report and assess the magnitude of heterogeneity. Random effects model was used in instances where I^2^ was more than 40% [25]. A P-value of less than 0.05 was considered statistically significant. Publication bias was also assessed using the Egger’s test.

## Results

**Fig 1:**
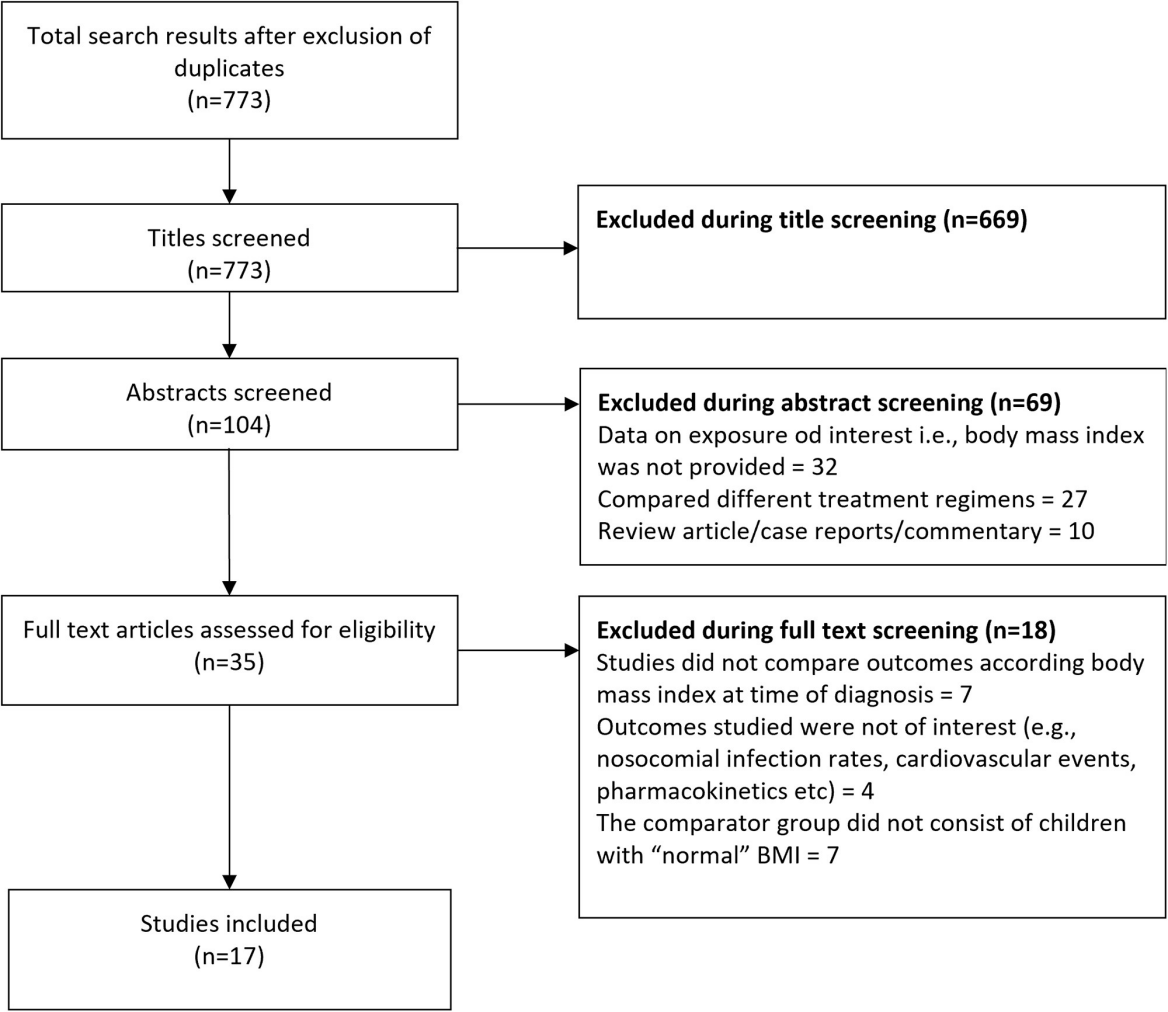
Selection process of the studies included in the review.

Database searching revealed 773 unique citations (Fig 1).

Of them, 669 studies were removed after the title screening, and additional 69 studies were removed after the abstract screening. The remaining 35 papers underwent detailed full-text review. Finally, 17 were included in the analysis [26–42].

Of these, 11 were prospective cohort studies and 6 were retrospective data analyses. Seven studies were conducted in the USA. One study each was conducted in Mexico, Canada, United Kingdom, Brazil, China and Thailand. Finally, four multi-centre studies were included. Ten studies investigated pediatric acute lymphoblastic leukemia (ALL) patients, five investigated pediatric myeloid leukemia (AML) patients, and two investigated a mixed cohort, including patients with ALL, AML, chronic myeloid leukemia, or myelodysplastic syndrome) (Table 1). The quality of the included studies was judged to be good (Table 1).

**Table 1:** Characteristics of the studies included in the meta-analysis.

Author (year of publication)	Study design	Country	Participant characteristics	Operational definition	Sample size	New castle Ottawa score
Egnell et al (2020) [26]	Prospective cohort	Multicentric (Nordic countries- Sweden, Norway, Denmark, Finland, Iceland)	All participants had ALL (acute lymphoblastic leukaemia); Participant age between 2–17.9 years; median age of 5 years; 55% males; Immunophenotype- B cell lineage (88%); hematopoietic stem cell transplantation not done in majority (88%); low risk cytogenetics (hyper diploidy; t 12; 21)	Body mass index (kg/m^2^) categorized using International Obesity Task Force (IOTF) childhood BMI cut-offs: Underweight: <17 Normal: 17 to 25 Overweight: 25 to 30 Obese: ≥30	2558	7
Martin-Trejo et al (2017) [27]	Prospective cohort	Mexico	All had ALL (acute lymphoblastic leukaemia); Majority (68%) aged between 2–10 years; median age of 6 years; 55% males; Immunophenotype- B cell lineage (89%); majority with low risk cytogenetics (58%)	Normal, underweight, overweight and obese categorized according to WHO/CDC criteria	782	7
Lohmann et al (2019) [28]	Prospective cohort	Multicentric	All had AML (acute myeloid leukaemia); Age range of 2–17 years; median age of 10 years; 53% males; majority with no stem cell transplantation (74%)	BMI categorized as per WHO Child growth standards: Underweight: <−2 SD, Healthy weight: −2 to +2 SD for age 2‐5 and −2 to +1 SD for age 6‐17, Overweight: >2‐3 SD for age 2‐5 and >1‐2 SD for age 6‐17, Obesity: >3 SD for age 2‐5 and >2 SD for age 6‐17.	867	7
Eissa et al (2017) [29]	Prospective cohort	USA	All had ALL (acute lymphoblastic leukaemia); Majority (72%) aged between 2 to <10 years; Age range of 2–18 years; 57% males; Immunophenotype- B cell lineage (83%); majority with low to standard risk (90%)	Underweight: BMI below the 5th percentile, Normal: BMI in or above the 5th percentile but below the 85^th^ percentile, Overweight: BMI in or above the 85th percentile but below the 95th percentile, Obese: BMI in or above the 95th percentile	373	8
Aldhafiri et al (2014) [30]	Retrospective (Secondary analysis of data)	UK	All had ALL (acute lymphoblastic leukaemia); Age range of 2–14.9 years; Median age of 4.9 years; males (77%)	Obesity defined as BMI of 30 kg/m2; Underweight defined as BMI <18.5 kg/m2	917	7
Hijiya et al (2006) [31]	Retrospective review of records	USA	All had ALL (acute lymphoblastic leukaemia); participants aged 1.01–18.8 years; 50% males; Median age of around 6 years; Immunophenotype- B cell lineage (90%)	Underweight: BMI-for-age ≤ 10th percentile; normal: > 10th and < 85th percentile; overweight: ≥85th percentile and < 95th percentile; obese: BMI-for-age ≥ 95th percentile	621	7
Orgel et al (2014) (A) [32]	Prospective cohort	USA	All had ALL (acute lymphoblastic leukaemia); Mean participants aged 10.2 years; 60% males; Immunophenotype- Pre-B cell lineage	BMI percentile for children underweight (< 5^th^ percentile) or obese (≥ 95^th^ percentile), normal (5^th^ to 95^th^ percentile) as per Centers for Disease Control and Prevention (CDC) guidelines	2008	8
Orgel et al (2014) (B) [33]	Prospective cohort	USA	All had ALL (acute lymphoblastic leukaemia); Mean participants aged 8.2 years; Age range of 1.2 to 18.9 years; 51% males; Immunophenotype- Pre-B cell lineage; standard risk (56%); 80% Hispanic	Overweight (85% to 94% inclusive); obese (≥95th percentile); lean (below the 85th percentile) as per Centers for Disease Control and Prevention (CDC) guidelines	198	8
Canner et al (2013) [34]	Prospective cohort within a clinical trial	USA	All had AML (acute myeloid leukaemia); Mean participants aged 6.9 years; normal cytogenetics (21%);; standard to low risk (90%); matched family donor transplant not received in majority (85%)	Body mass index (BMI) calculated for children 2 years of age and older, whereas weight-for-height (WFH) ratio for those younger than 2 years of age as per Centers for Disease Control and Prevention (CDC) guidelines; underweight (< 5^th^ percentile), obese (≥ 85^th^ percentile), normal (5^th^ to 84^th^ percentile)	1840	7
Inaba et al (2012) [35]	Prospective cohort within a clinical trial	USA	All had AML (acute myeloid leukaemia); Age range of 2–20 years; around 50% males; matched family donor transplant not received in majority; majority with normal cytogenetics	Based on CDC weight status and BMI percentiles: underweight, < 5th percentile; healthy weight, 5th to 85th percentile; overweight, 85th to 95th percentile; obese, ≥ 95th percentile.	314	7
Lange et al (2008) [36]	Prospective cohort within a clinical trial	USA	All had AML (acute myeloid leukaemia); median age of 9.5 years; around 52% males; majority with normal (22%) or t(8;21) (16%) cytogenetics; standard risk (61%)	BMI percentiles: obese (>95 percentile), middle weight (10–94% percentile), and underweight (<10% percentile)	901	8
Aplenc et al (2014) [37]	Retrospective review of database	Multicentric (USA, Canada, Europe, Asia, Middle East)	Participants with mixed diagnosis; majority with ALL (45%) and AML (30%); others include chronic myeloid leukaemia and myelodysplastic syndrome; age range of 2–19 years; 59% males	Underweight: <5th percentile; normal: 25th to 85th percentile; overweight: 86th to 95th percentile; and obese: >95th percentile	3687	8
Bulley et al (2007) [38]	Prospective cohort	Canada	Participants with mixed diagnosis; majority with ALL (39%) and AML (20%); age range of 2–19 years; median age of 8.4 years; 61% males	Obesity was defined as BMI ≥95th percentile; normal BMI >10th to <95th percentile, underweight as BMI ≤10^th^ percentile using the Centers for Disease Control and Prevention (CDC) guidelines	325	7
Gelelete et al (2011) [39]	Retrospective analysis of data	Brazil	Participants with ALL; majority with age under 10 years; 53% males; Immunophenotype- B cell lineage (78%)	Overweight was defined as a BMI above 1 SD and obesity as BMI above +2 SD of *z*-score for age and sex as per WHO Child Growth Standards	181	6
Surapolchai et al (2012) [40]	Retrospective analysis of data	Thailand	Participants with ALL; majority with age under 9 years (79%); 60% males; Immunophenotype- B cell lineage (89%); low to standard risk (80%)	BMI-for-age ≥ 95th percentile using the Centers for Disease Control and Prevention (CDC) guidelines	94	6
Hu (2023) [41]	Retrospective cohort	China	All participants had ALL (acute lymphoblastic leukaemia); median age of 5.7 years; male (62%); median follow up of 6.3 years	Underweight: BMI below the 5th percentile, Normal: 5^th^ to 85^th^ percentile, Overweight: > 85th percentile to <95th percentile, Obese: ≥ 95th percentile	1437	8
Iijima (2022) [42]	Prospective cohort	Multicentric	All participants had AML (acute myeloblastic leukaemia); majority had age between 0 to 10 years (52%); male (51%); follow up of 5 years	Underweight: BMI below the 5th percentile, Normal: 5^th^ to 85^th^ percentile, Overweight: > 85th percentile to <95th percentile, Obese: ≥ 95th percentile	227	8

BMI was the primary exposure. Included studies differed in the BMI cut-offs adopted for defining underweight, normal and obese children. Specific criteria used by the individual studies have been presented in Table 1. The different criteria were comprised of guidelines by the International Obesity Task Force (n = 1 study), z- score based approach (n = 1 study), BMI percentile-based approach (n = 12 studies) and use of WHO/CDC criteria (n = 3 studies). The variability in the cut-offs for categorizing underweight, normal, overweight and obese children was detected even in studies that used the percentile-based approach.

### Body mass index (BMI) at diagnosis and overall mortality

**Fig 2:**
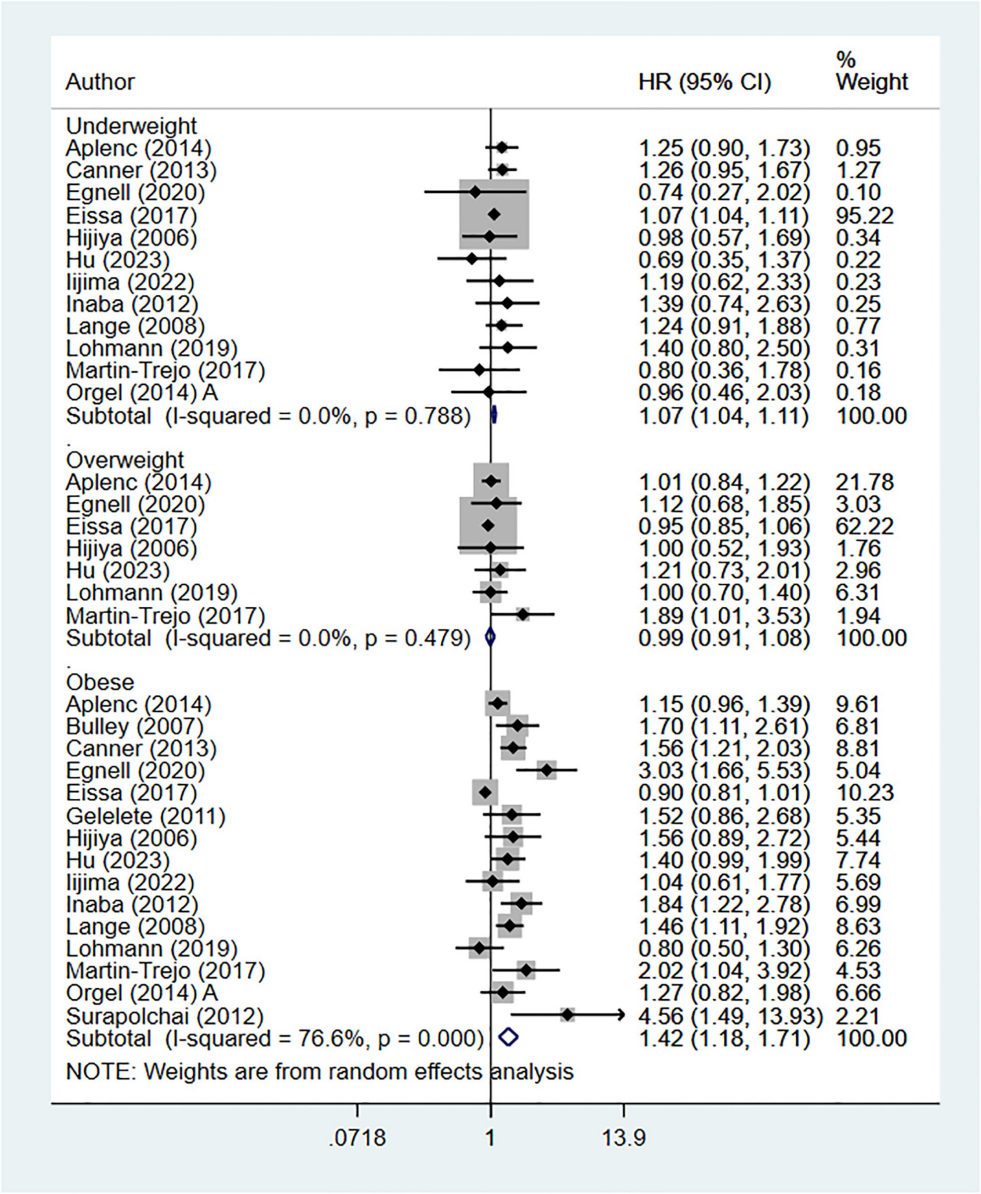
Body mass index at diagnosis and risk of overall mortality.

Underweight (HR 1.07, 95% CI: 1.04, 1.11; I^2^ = 0.0%, N = 12) or obese (HR 1.42, 95% CI: 1.18, 1.71; I^2^ = 76.6%, N = 15) pediatric leukemia patients showed greater mortality risk compared to children with normal BMI. However, no difference in mortality risk was noted in overweight children (HR 0.99, 95% CI: 0.91, 1.08; I^2^ = 0.0%, N = 7) (Fig 2).

We noted that for the comparison between underweight and normal BMI children, around 95% of the weight was given to the study by Eissa et al (2017) [29]. Therefore, we repeated the analysis after excluding this study. Our findings still indicated an increased risk of mortality (HR 1.17, 95% CI: 1.01, 1.35; I^2^ = 0.0%, N = 11) (S1 Fig). Similarly, we found that for the comparison between overweight and normal BMI children, around 60% of the weight was given to the study by Eissa et al. A sensitivity analysis that excluded this study did not detect difference in the mortality risk in overweight children (HR 1.06, 95% CI: 0.92, 1.23; I^2^ = 0.0%, N = 6) (S1 Fig).

Egger’s test did not detect the presence of publication bias (P = 0.40 for underweight category; P = 0.27 for overweight category; P = 0.64 for obese category). Subgroup analysis showed that underweight [ALL: HR 1.07, 95% CI: 1.02, 1.11; AML: HR 1.28, 95% CI: 1.03, 1.55] and obese [ALL: HR 1.67, 95% CI: 1.11, 2.55; AML: HR 1.41, 95% CI: 1.07, 1.83] children with ALL and AML both showed elevated risk of mortality compared to children with normal BMI (S2 Table).

### Body mass index (BMI) at diagnosis and event-free mortality

**Fig 3:**
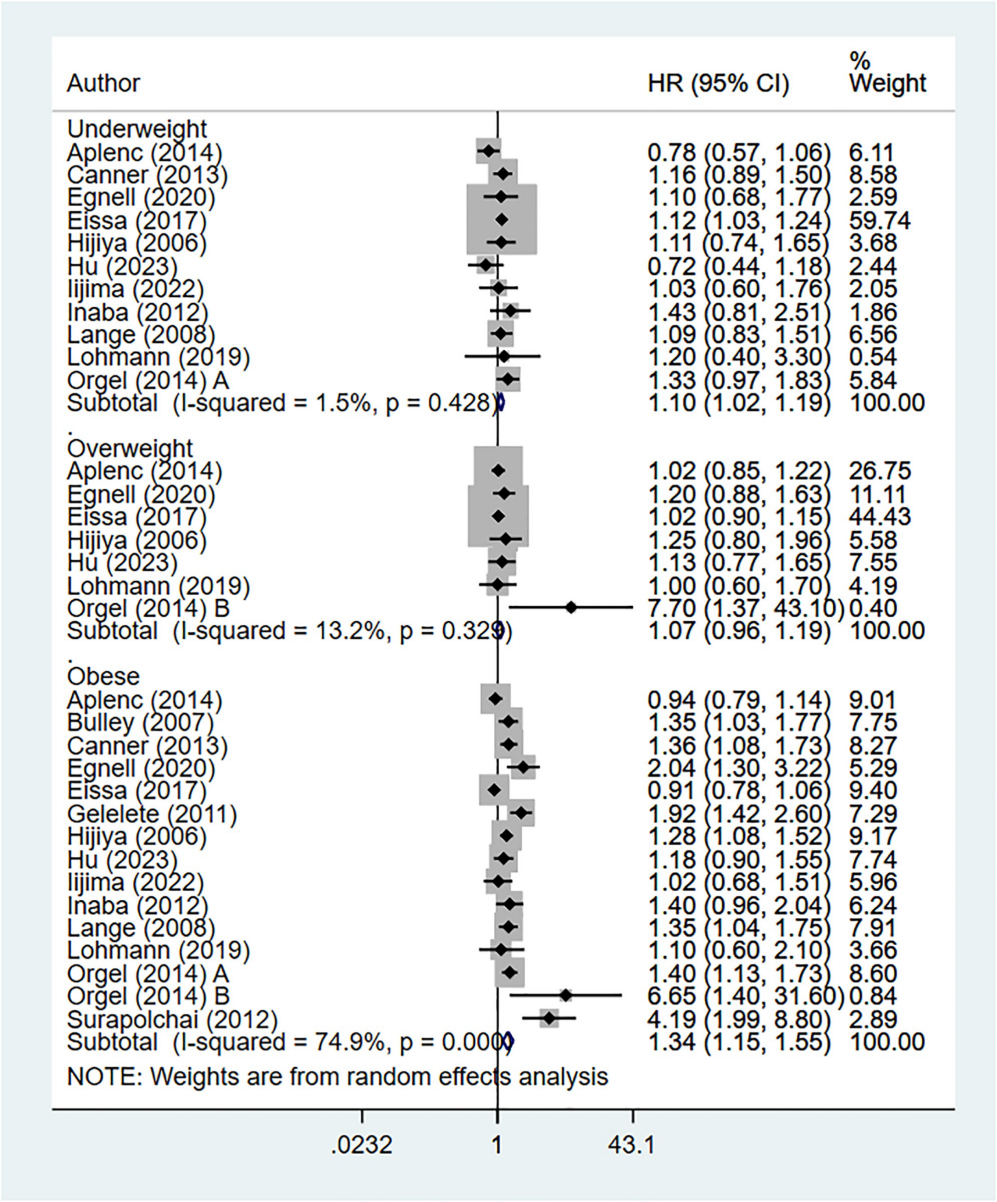
Body mass index at diagnosis and event free survival.

Underweight (HR 1.10, 95% CI: 1.02, 1.19; I^2^ = 1.5%, N = 11) and obese (HR 1.34, 95% CI: 1.15, 1.55; I^2^ = 74.9%, N = 15) children showed lower rates of event-free survival compared to normal BMI children (Fig 3).

No significant difference in EFS was noted between overweight and normal BMI children (HR 1.07, 95% CI: 0.96, 1.19; I^2^ = 13.2%, N = 7). No evidence for publication bias was noted (P = 0.63 for underweight category; P = 0.59 for overweight category; P = 0.36 for obese category).

For analysis related to underweight children, around 60% of the weight was allocated to study by Eissa et al (2017) [29]. We did a re-analysis after excluding this study and noted that the association with EFS was not statistically significant (HR 1.07, 95% CI: 0.94, 1.21; I^2^ = 7.9%, N = 10) (S2 Fig). Similarly, we found that for the comparison between overweight and normal BMI children, around 45% of the weight was given to the study by Eissa et al. On doing a sensitivity analysis after excluding this study, no statistical difference was found in the risk of event free mortality in overweight children (HR 1.12, 95% CI: 0.95, 1.32; I^2^ = 20.9%, N = 6) (S2 Fig). Subgroup analysis showed that underweight [ALL: HR 1.13, 95% CI: 1.05, 1.24; AML: HR 1.16, 95% CI: 1.03, 1.37] and obese [ALL: HR 1.62, 95% CI: 1.20, 2.23; AML: HR 1.35, 95% CI: 1.14, 1.56] children with ALL and AML both showed lower EFS rates compared to children with normal BMI (S2 Table).

### Body mass index (BMI) at diagnosis and risk of relapse

**Fig 4:**
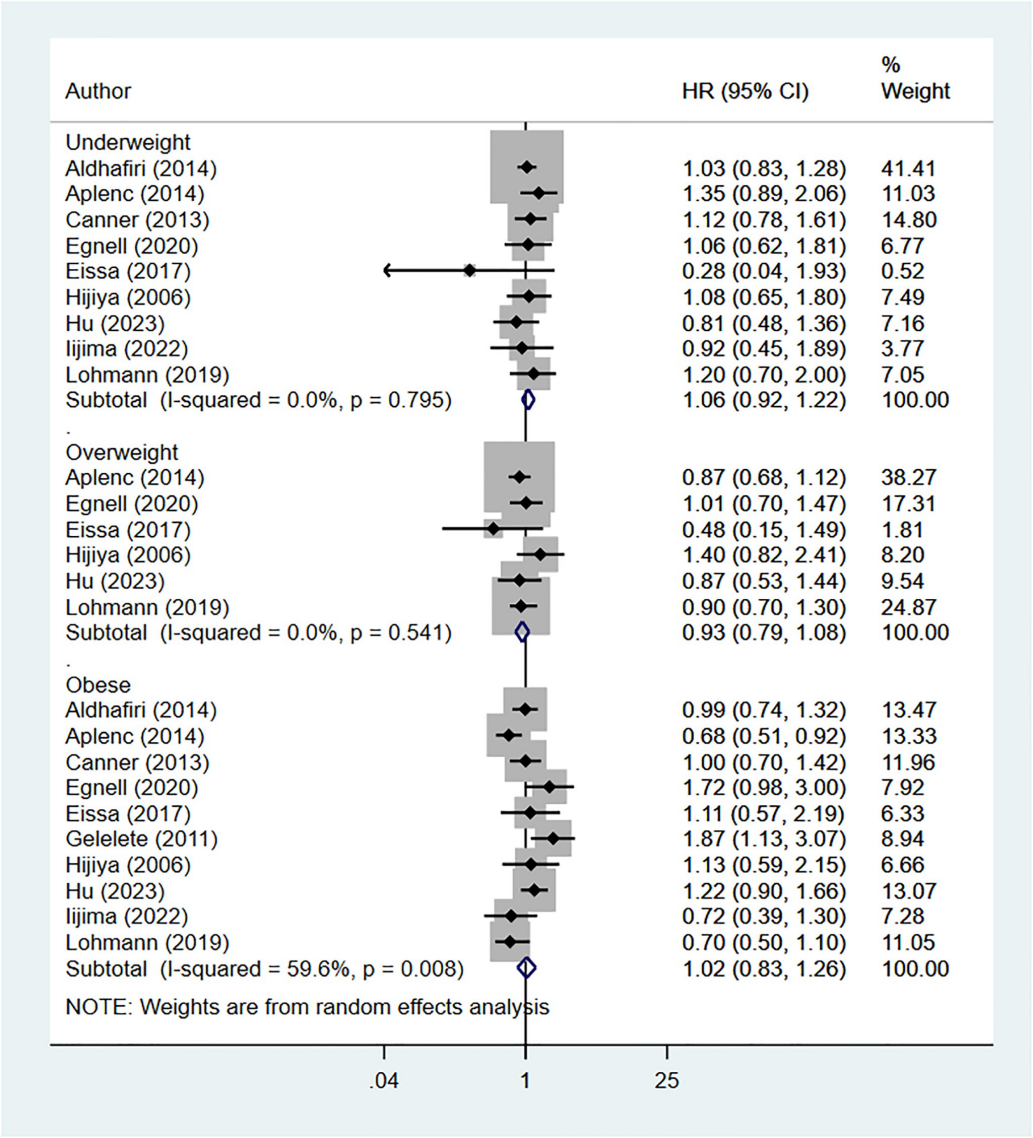
Body mass index at diagnosis and risk of relapse.

Risk of relapse was comparable in underweight (HR 1.06, 95% CI: 0.92, 1.22; I^2^ = 0.0%, N = 9), overweight (HR 0.93, 95% CI: 0.79, 1.08; I^2^ = 0.0%, N = 6), obese (HR 1.02, 95% CI: 0.83, 1.26; I^2^ = 59.6%, N = 10), and normal-BMI pediatric leukemia patients (Fig 4).

We did not find evidence of publication bias (P = 0.20 for underweight category; P = 0.31 for overweight category; P = 0.77 for obese category). Subgroup analysis showed that underweight, overweight, and obese children with ALL or AML were not at an increased risk of relapse, compared to children with normal BMI (S2 Table).

## Discussion

Our review found that underweight or obese pediatric leukemia patients had higher risk of mortality and poor EFS compared to children with normal BMI. There was no increased risk of mortality or poor EFS among patients who were overweight. Subgroup analysis showed that this effect of underweight and obese status on the risk of mortality and EFS was detected in both ALL and AML patients. Our results emonstrated that the risk of relapse was statistically similar across all BMI categories (underweight, overweight and obese), when compared to normal BMI. Our findings are similar to the earlier two reviews that also noted that higher BMI is associated with increased risk of mortality and lower EFS [21, 22]. However, earlier reviews did not clarify the operational definition of “higher BMI” and had variability in comparison groups among different included studies. For some, the comparator was normal BMI, while for others it was a combination of normal and underweight BMI. Further, previous reviews did not consider other BMI categories as a “exposure of interest”. In contrast, current meta-analysis showed that both high BMI (i.e., obesity) and underweight status were risk factors for poor outcomes.

The observed association of obesity with poor outcomes may be explained by several mechanisms. Obesity may impact pharmacokinetics and/or pharmacodynamics of the chemotherapeutic drugs, particularly those that are lipophilic [43, 44]. This may, in turn, affect the efficacy of these drugs and consequently, the outcomes. Obese children with ALL are likely to be at higher risk for treatment-related toxicities, such as steroid-induced hyperglycemia, asparaginase-induced pancreatitis, hepatic steatosis and osteonecrosis [45, 46]. This can easily limit the effective administration of chemotherapy and can force dose reductions. All these factors might contribute towards decreasing the probability of survival and/or EFS in children with high BMI. Also, obesity leads to a stage of chronic inflammation that may provide conducive milieu for tumour growth [47, 48]. Childhood obesity has been shown to be associated with quite a few comorbid conditions which may further reduce the quality of life [49].

Prior studies have indicated that underweight patients are at increased risk of cancer recurrence and that underweight status increases the risk of mortality in cancer patients due to infection and associated sepsis [17, 50, 51]. We did not test the possibility of increased risk of infection and sepsis in underweight children but do believe that future studies should explore the BMI-infection-mortality pathway in pediatric cancer patients. Studies have also shown that there may be an increased risk of recurrence and metastasis in patients who are undernourished due to the increased tumour aggressiveness [17, 18]. Underweight children may experience compromised immune function and reduced physiological reserves, making them more susceptible to the adverse effects of aggressive cancer treatments. There have been suggestions that sub-optimal nutritional status might lead to an increased toxicity and possibly, a decreased response to chemotherapy [19, 20].

Both underweight and obese children may experience delays in diagnosis and initiation of treatment. Underweight children might be more vulnerable to delayed diagnosis due to atypical or less noticeable symptoms, while obesity may contribute to diagnostic challenges, masking symptoms or complicating the interpretation of laboratory results. Psychosocial factors might also play a role. Underweight children may face socioeconomic disparities and limited access to healthcare, impacting timely diagnosis and intervention. On the other hand, obese children might encounter stigma or bias in healthcare settings, potentially influencing the quality of care they receive.

We do acknowledge that there are certain limitations of this review. A noteworthy limitation is that most studies were retrospective and therefore, it is possible that some of the important variables or confounders were not adjusted in the analysis. This may make the reported effect sizes in individual studies biased. Majority of the studies failed to comprehensively report on the treatment protocols adopted for leukemia and therefore, it was not possible to elucidate how the treatment modified the observed association between BMI and the outcomes. In the included studies, the BMI was categorized into underweight, normal, overweight and obesity using different guidelines. This could have led to heterogeneity in the findings. Although we included studies from a wide geographical location, we were unable to explore the granularity of different protocols used globally. This was mainly due to the wide variation in the protocols used. The value of our analysis would have improved if we could have related the findings with the protocols (and doses of various drugs) employed in the included studies across the various country settings.

## Conclusion

Based on the findings of this meta-analysis, we conclude that underweight or obese children with ALL and AML leukemia tend to have an increased risk of poor survival outcomes. Future studies should explore the underlying mechanisms that lead to poor outcomes to identify potential areas of intervention. Studies should also explore the effect of interventions to address undernutrition and obesity in pediatric leukemia patients on the disease outcome.

## Supporting information

S1 Table Search strategy for identification of studies to be included in the review. (DOCX)

S2 Table Findings of the subgroup analysis based on the type of pediatric leukaemia. (DOCX)

S1 Fig Sensitivity analysis for overall survival, after excluding the study by Eissa et al (2017). (TIF)

S2 Fig Sensitivity analysis for event free survival, after excluding the study by Eissa et al (2017). (TIF)

S1 Checklist PRISMA 2020 checklist. (DOCX)
