# Supplementary material for: Impact of body mass index at diagnosis on outcomes of pediatric acute leukemia: A systematic review and meta-analysis
Source: PLoS One. 2024 May 6;19(5):e0302879. doi: 10.1371/journal.pone.0302879 (PMC11073705; doi:10.1371/journal.pone.0302879)
Supplement: S1 Table — (DOCX) [file pone.0302879.s001.docx]

**S1 Table. Search strategy for identification of studies to be included in the review**

| No. | Details |
| --- | --- |
| #1 | acute leukaemia OR acute lymphoblastic OR acute myeloid OR ALL OR AML |
| #2 | obesity OR overweight OR underweight OR body weight OR body mass index OR BMI OR body size OR weight |
| #3 | mortality OR survival OR death OR prognosis OR relapse OR progression OR recurrence OR remission OR disease-free survival OR event-free survival |
| #4 | paediatric OR children |
| #5 | #1 AND #2 AND #3 AND #4 |
| #6 | Addresses[ptyp] OR Autobiography[ptyp] OR Bibliography[ptyp] OR Biography[ptyp] OR pubmed books[filter] OR Case Reports[ptyp] OR Congresses[ptyp] OR Consensus Development Conference[ptyp] OR Directory[ptyp] OR Duplicate Publication[ptyp] OR Editorial[ptyp] OR Systematic reviews OR Meta analysis OR Festschrift[ptyp] OR Guideline[ptyp] OR In Vitro[ptyp] OR Interview[ptyp] OR Lectures [ptyp] OR Legal Cases[ptyp] OR News[ptyp] OR Newspaper Article[ptyp] OR Personal Narratives [ptyp] OR Portraits[ptyp] OR Retracted Publication[ ptyp] OR Twin Study[ptyp] OR Video-Audio Media[ptyp] |
| #7 | #5 NOT #6 |
