# Supplementary material for: Impact of body mass index at diagnosis on outcomes of pediatric acute leukemia: A systematic review and meta-analysis
Source: PLoS One. 2024 May 6;19(5):e0302879. doi: 10.1371/journal.pone.0302879 (PMC11073705; doi:10.1371/journal.pone.0302879)
Supplement: S2 Table — (DOCX) [file pone.0302879.s002.docx]

**S2 Table. Findings of the subgroup analysis based on the type of Pediatric leukaemia**

| **Outcomes of interest** | | **Acute lymphoblastic leukaemia (ALL)** | **Acute myeloid leukaemia (AML)** |
| --- | --- | --- | --- |
|  |  | Pooled Hazard ratio (HR) with 95% CI | |
| Overall survival | Underweight | 1.07 (1.02, 1.11) *  N=6; I^2^=0.0% | 1.28 (1.03, 1.55) *  N=5; I^2^=0.0% |
|  | Overweight | 1.08 (0.82, 1.42)  N=5; I^2^=37.3% | 1.00 (0.71, 1.41)  N=1 |
|  | Obese | 1.67 (1.11, 2.55) *  N=8; I^2^=81.3% | 1.41 (1.07, 1.83) *  N=5; I^2^=60.2% |
| Event free survival | Underweight | 1.13 (1.05, 1.24) *  N=5; I^2^=2.0% | 1.16 (1.03, 1.37) *  N=5; I^2^=0.0% |
|  | Overweight | 1.17 (0.91, 1.53)  N=6; I^2^=51.8% | 1.00 (0.59, 1.68)  N=1 |
|  | Obese | 1.62 (1.20, 2.23) *  N=8; I^2^=83.5% | 1.35 (1.14, 1.56) *  N=5; I^2^=1.7% |
| Relapse | Underweight | 1.03 (0.84, 1.23)  N=5; I^2^=0.0% | 1.14 (0.86, 1.53)  N=3; I^2^=0.0% |
|  | Overweight | 1.06 (0.70, 1.57)  N=4; I^2^=30.5% | 0.90 (0.66, 1.23)  N=1 |
|  | Obese | 1.27 (0.97, 1.71)  N=6; I^2^=37.1% | 0.85 (0.61, 1.19)  N=3; I^2^=43.1% |

*Statistical significance at P<0.05
