# Supplementary figures and images for: Impact of body mass index at diagnosis on outcomes of pediatric acute leukemia: A systematic review and meta-analysis
Source: PLoS One. 2024 May 6;19(5):e0302879. doi: 10.1371/journal.pone.0302879 (PMC11073705; doi:10.1371/journal.pone.0302879)

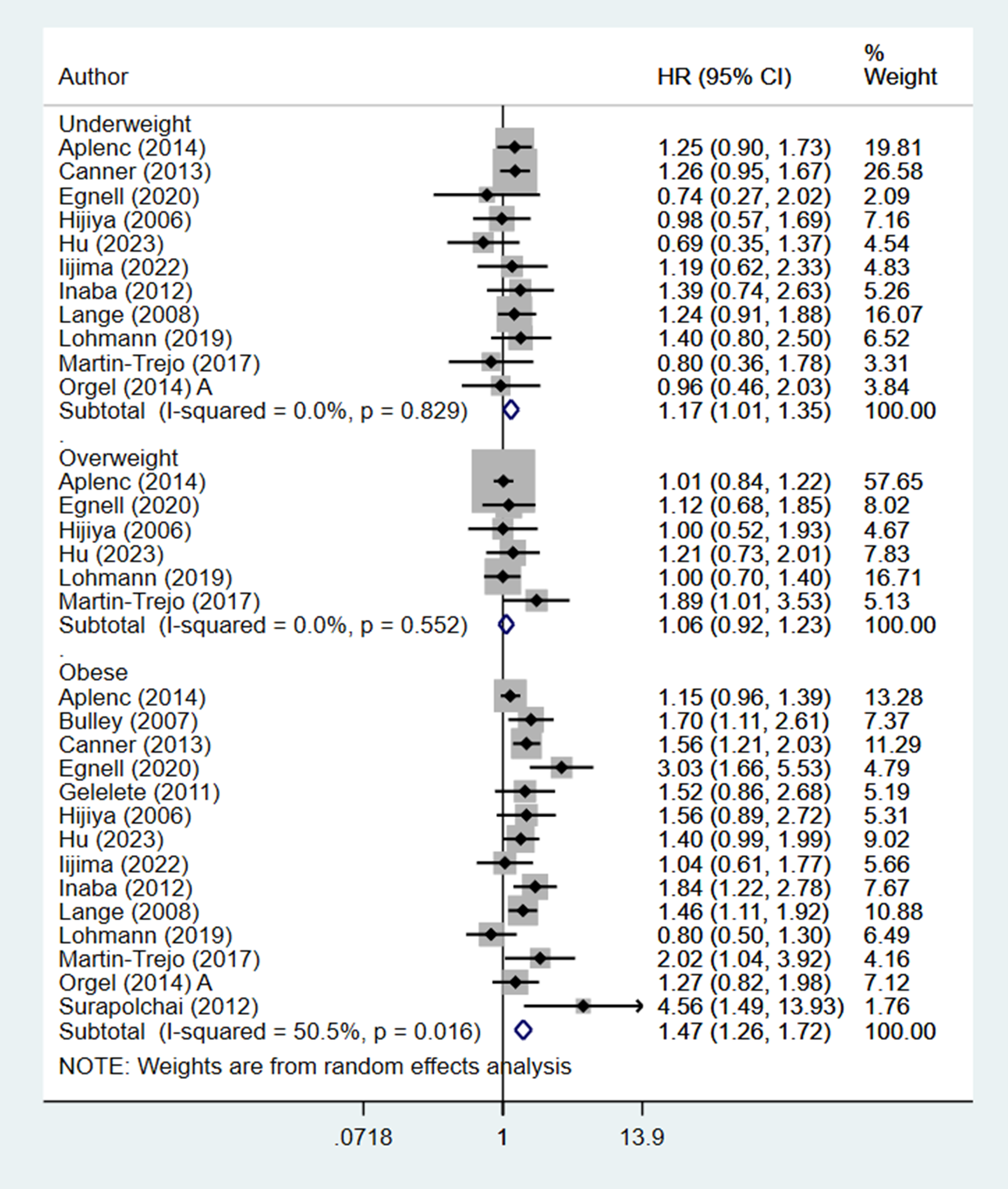

Supplement: S1 Fig — (TIF) [file pone.0302879.s003.tif]

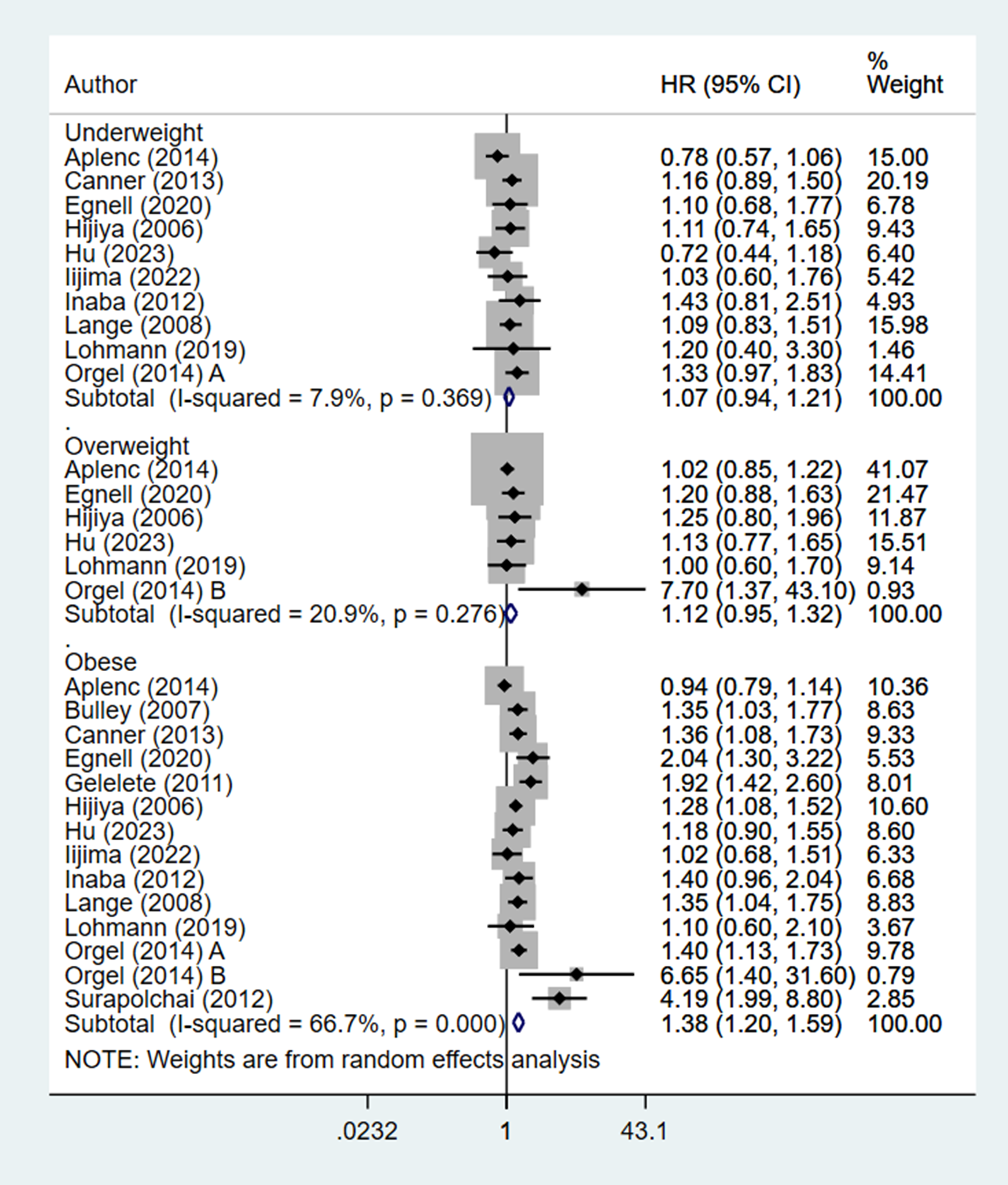

Supplement: S2 Fig — (TIF) [file pone.0302879.s004.tif]
